# Supplementary material for: Learning curve and surgical outcome of robotic assisted colorectal surgery with ERAS program
Source: Sci Rep. 2022 Nov 29;12:20566. doi: 10.1038/s41598-022-24665-w (PMC9709162; doi:10.1038/s41598-022-24665-w)
Supplement: Supplementary file 1 — Supplementary Information 1. [file 41598_2022_24665_MOESM1_ESM.zip › LSC Raw data-Ñ[▒K/137/P_ñw╝╨░O▒Kñσ.pdf]

[redacted]

Pathologic diagnosis:
1. Sigmoid colon, laparoscopic anterior resection --- Status post polypectomy with residual intramucosal adenocarcinoma, moderately differentiated. The bilateral cut ends and mesocolic lymph nodes (0/10) are free of tumor.
2. IMA root, dissection --- Unremarkable lymph node (0/1).
3. Colon, cut end, distal, resection --- Unremarkable.

Ancillary study for diagnosis:
1. Deep cut done for section E.
2. Recut and IHC stain for CD34 (highlights vessels) done for section F.

Prognostic and predictive factor:
(combined with report S1018445)
1. Tumor size: 0.6 cm.
2. Depth of invasion: Subucosa (presented in previous polypectomy specimen, S1018445).
3. Histologic grade: G2: Moderately differentiated.
4. Angiolymphatic invasion: Not identified.
5. Perineural invasion: Not identified.
6. Tumor deposits (discontinuous extramural extension): Not identified.
7. Macroscopic tumor perforation: Not identified.
8. Surgical Margins: a) Proximal Margin: Uninvolved by invasive carcinoma, distance of tumor from margin: 11 cm. b) Distal Margin: Uninvolved by invasive carcinoma, distance of tumor from margin: 3 cm. c) Radial or Mesenteric Margin: Uninvolved by invasive carcinoma, distance of tumor from margin: 4 cm.
9. Lymph node status: Uninvolved (0/11).
10. Extranodal involvement: Not identified.
11. Tumor regression grading S/P CCRT: No known presurgical therapy.
12. Type of polyp in which invasive carcinoma arose: Not identified.
13. Additional pathologic findings: Not identified.
14. Pathological TNM Stage: pT1N0 (According to the eighth edition, American Joint Committee on Cancer Staging Guidelines for Tumors).
15. TNM descriptors: Not applicable.

Gross description:
The specimen consists of 1) a segment of colon, 15 cm in length and 5 cm in proximal cut end circumference, 6 cm in distal circumference, and mesocolic soft tissue. A gray tan soft polypoid tumor, 0.7x0.7 cm, is noted, 3 cm to the distal cut end. On cross section, the tumor invades to the submucosa with 4 cm to the radial margin. The both cut ends are free of tumor grossly. 2) IMA root, a piece of tan yellow soft tissue, 4x3x2 cm. 3) distal cut end, a piece of tan mucosa-covered soft tissue, 3x2x1 cm. Representative part for section: A) specimen 2 B) specimen 3 C) proximal cut end D) radial margin E) mucosa F-G) tumor H-K) lymph node.

Note:
This case has been peer reviewed by two doctors.
#T-59470_2 #M-81403_2 2 1105 B0000F
#T-C4560_2 #M-09183_2 0 1105 000000

已電子簽章

敬告：本單僅供醫師參考使用，不具任何申請效力！！
請病患妥為保管，若不慎遺失需自負個人資料外洩責任！
